# Predicting risks and benefits of treatment with aspirin in the acute stage of ischaemic stroke: an analysis of 3 large randomised controlled trials

**DOI:** 10.1186/1745-6215-14-S1-P117

**Published:** 2013-11-29

**Authors:** Douglas Thompson, Gordon Murray, William Whiteley

**Affiliations:** 1 Edinburgh MRC Hub for Trials Methodology Research, University of Edinburgh, Edinburgh, UK; 2 Division of Clinical Neurosciences, University of Edinburgh, Bramwell Dott Building, Western General Hospital, Edinburgh, UK

## Background

Aspirin reduces the absolute risk of death or dependence following an acute ischaemic stroke. An associated increase in risk of haemorrhage may cause considerable harm. We hypothesised that patients at a high predicted risk of further thrombosis or a low risk of haemorrhage would experience greater absolute benefit from aspirin. In addition we explored the assumption that absolute benefit increases with baseline risk.

## Methods

We applied formal prediction methods to the three largest randomised trials of aspirin in patients with acute ischaemic stroke. We developed new prediction models for early events (14 day thrombosis and haemorrhage) and for long term functional outcome (six month death or dependence) and internally evaluated their performance. We calculated the absolute risk reduction of death or dependence with aspirin within quarters of predicted patient risk from early events across trials and pooled the results using random effects meta-analysis.

## Results

Simple prediction models discriminated early events poorly (AUROCC 0.56 and 0.60) but were moderate at discriminating long term death or dependence (AUROCC 0.77). There was no evidence of greater benefit or of harm from aspirin across the sixteen defined subgroups of predicted risk nor was there any evidence that absolute benefit increased linearly with baseline risk. The best estimate of the effect of aspirin was the overall absolute risk reduction of death or dependence of 1% (95%CI: 0% to 2%) in all risk groups.

## Conclusions

We found no evidence to support targeting aspirin to acute ischaemic stroke patients with a high predicted risk of thrombosis or a low predicted risk of haemorrhage. The modest absolute benefit of aspirin was similar across predicted patient risk of death or dependence.

